# The Effect of Chip Binding on the Parameters of the Case-Hardened Layer of Tooth Surfaces for AMS 6308 Steel Gears Processed by Thermochemical Treatment

**DOI:** 10.3390/ma14051155

**Published:** 2021-03-01

**Authors:** Robert Fularski, Ryszard Filip

**Affiliations:** 1 Department of Gears, Pratt & Whitney Rzeszów S.A., Hetmańska 120, 35-078 Rzeszów, Poland; 2 Department of Materials Science, Faculty of Mechanical Engineering and Aeronautics, Ignacy Łukasiewicz Rzeszów University of Technology, Powstańców Warszawy 12, 35-959 Rzeszów, Poland

**Keywords:** skiving, cutting, circumferential peeling, chip, carburized case, case hardness, gears, surface layer, welding, binding

## Abstract

The following article describes influence of pressure welded or bound chips to the gear tooth flank and/or the tooth root on a carburized case and surface layer hardness of Pyrowear 53 steel gears, machined by Power Skiving method. This paper is focused only on one factor, the chips generated while forming gear teeth by power skiving, which could result in local changes in the carburized case parameters as a negatively affecting point of mechanical performance of the carburized case. The chips, due to the specifics of the power skiving process and the kinematics of tooth forming, could be subject to the phenomena of pressure welding or binding of chips to the tooth. During the carburizing stage of the downstream manufacturing processes, the chips form a diffusion barrier, which ultimately could result in localized changes in the carburized case. This work was an attempt to answer the question of how and to what extent the chips affect the case hardening. Performed simulations of chips by a generating cupper “spots”, mentioned in the study, represent a new approach in connection with minimization of errors, which could appear during carbon case depth and case hardness analysis for typical chips, generated during the machining process—assurance that a complete chip was bound to the surface. Hardness correlation for zones, where the chip appears with areas free of chips, gives simple techniques for assessment. Performed tests increased the knowledge about the critical size of the chip—1.5 mm, which could affect the case hardening. Obtained experimental test results showed that the appearance of chip phenomena on the gear tooth might have a negative impact on a carburized case depth and hardened layer.

## 1. Introduction

Despite intense technical progress, gears continue to be available in a broad range of different applications. This includes common household products, automotive products, aircraft gearboxes, and other applications in which the transmission of high power is important. Given their area of application, the gears used in aircraft gearboxes should feature a high quality of manufacturing, according to the stringent requirements related to gear tooth geometry and retention of the surface layer parameters, including carburizing case.

Materials used for their production must have resistance for: abrasion, contact stress on the surface layer, bending strength at the tooth base, and also require properties to withstand cyclic variable loads in the core. Heavy duty gears used in aviation gearboxes are usually made from high-strength alloy steels like AMS 6308 [1] (the chemical composition of Pyrowear^®^ 53 is presented in Table 1). The AMS 6308 is a relatively new steel originally prepared for military products, but for about a dozen years, has been increasingly used in civil aviation. Due to the growing of industry interest, it has become the subject of scientific considerations, too [1,2,3,4].

The properties of carburizing case of this steel may change not only by modification of the thermochemical processing parameters, but also as a result of machining, which includes both abrasive and cutting processes. It is also known that usage of quite new techniques, e.g., electro-pulsing, ultrasonic surface layer treatment presented by authors of publications [5,6,7,8], could improve the surface structure, mechanical properties, and microstructure, in some cases. However these technics cannot be directly applied to gear teeth surfaces improvement due to the specific geometric shape of gear teeth. While the phenomena which occur during machine cutting or abrasive machining process and their effect on the surface layer are widely discussed in the available references [9,10,11,12,13,14,15,16,17,18] and the industry norms applied in the assessment of microstructure, the question of machining prior to case carburizing of the surfaces to be exposed to considerable and dynamically variable loads is less extensively detailed in research work. However, it is known, that certain external factors related to machining could result in local changes in the carburized case parameters to a point capable of negatively affecting the mechanical performance of the carburized case.

This work is focused on one of the factors, the chips generated while forming gear teeth by power skiving [19,20], an increasingly popular tooth forming technique in the industry, capable of significantly improving machine cutting efficiency in comparison to the Fellows process, for example. 

**Figure 1:**
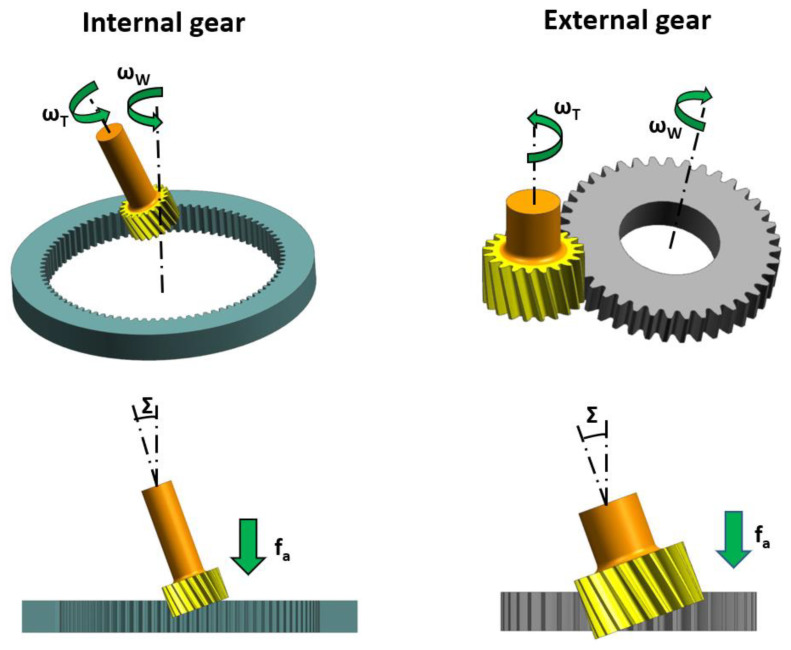
Basic kinematics of power skiving for internal and external gears.

**Figure 2:**
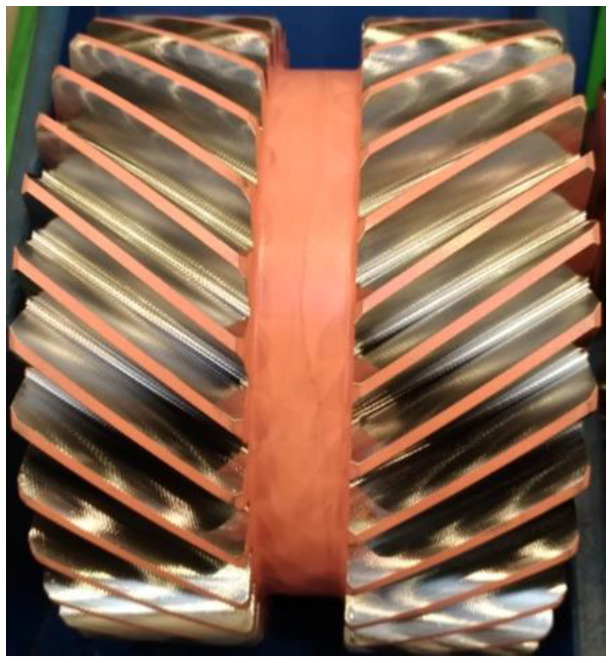
Double helical gear after power skiving machining.

Each sector of industry strives to optimize the manufacturing processes and reduce the production time at each step of the manufacturing cycle. In recent years, thanks to advances in technology, engineering and the drive for machines have created the opportunity for implementing a new method of gear tooth cutting in serial production, known as power skiving, or simply skiving. Power skiving was developed and patented by Julius Wilhelm von Pittler in 1910 [21,22], but due to its high processing and engineering requirements, which include high rigidity of the machine tool and the cutting tool holder, the high ratio of workpiece-to-tool spindle synchronization, and the required cutting tool durability, it was many years before power skiving became achievable for machine builders or the machine engineering industry. Advancements in various scientific disciplines have resulted in the development of new grades of steel, cutting tool coatings, and rigid machine tools combined with precision drive units and, in relation to the potential offered by the technology, rekindled interest in power skiving as a process capable of significantly improving machine cutting efficiency—for the forming of gear teeth. Skiving has certain unique requirements, which include the inclination angle of the tool spindle centerline to the workpiece centerline. In skiving, it is the angle that generates the cutting speed, the vector of which depends on the vectors of tangential velocity of the workpiece and the tangential velocity of the cutting tool. The dependencies of skiving kinematics, the specifics of skiving, and the requirements of skiving are discussed further by the authors of [19,20,21,22,23,24,25], for example. A significant factor which must always be considered in any approach to skiving is the inclination angle Σ (Figure 1), the optimum value of which is approximately 20°, for effective machining efficiency. Note, however, that this inclination angle cannot always be achieved due to the expected resultant geometry of the gear being processed. Given the specific nature and requirements of skiving, it is necessary at the tool design stage, especially concerning the cutting edges, to plan for all the expected phenomena related to chip formation and their removal from the cutting zone. As power skiving involves high rotational speeds, which translates into reduced gear manufacturing duration compared to the Fellows tooth forming process, the chips generated by the cutting feed cannot always be removed from the cutting zone due to the geometry of the gear being processed. A typical example of this involves internal tooth gears (Figure 1—Internal gear), where after chip formation during the cutting process, the chips are subjected to a centrifugal force which obstructs clearing of the cutting zone. The difficult removal of the chips from the cutting zone can also be observed in the processing of external tooth wheels, where the feed moves vertically upward and gravity causes the generated chips to move across the cutting zone. Figure 2 shows an example of the double helical gear cutting process. The process is split in two phases where cutting tool feed f_a_ (Figure 1, External gear) for each rim has an opposite direction. 

**Figure 3:**
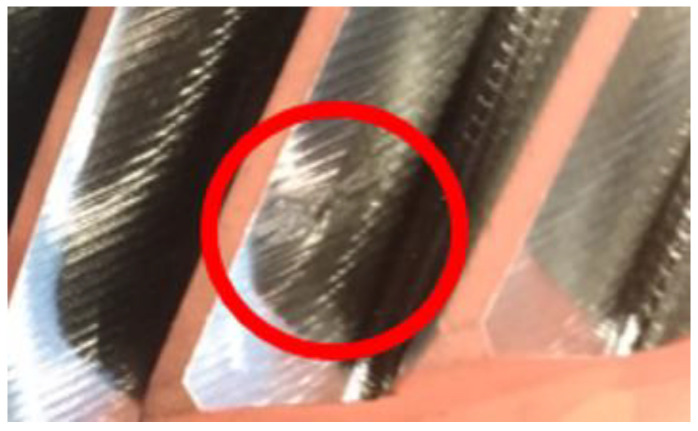
Bound chip on the tooth involute surface, after power skiving machining.

**Figure 4:**
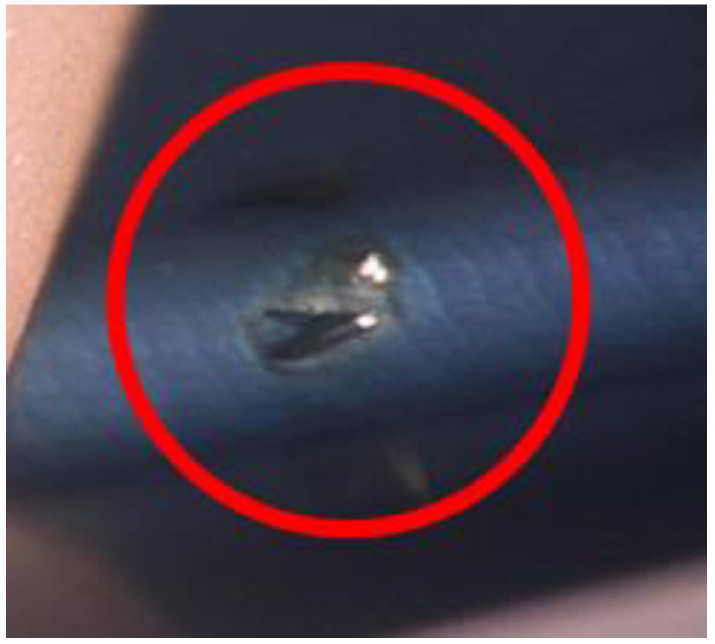
Bound chip on the tooth root, after power skiving machining.

When a chip moves between the cutting tool blade and the tooth being formed, unfavorable effects may result, such as fusion or binding of the chips to the tooth surfaces (Figure 3 and Figure 4). 

Given the potential for the effect to occur, as described in [19,20], for example, research was undertaken to determine the effect of binding a chip, generated during machining of power skiving method to tooth surfaces, seen as a specific foreign object, on the properties characteristic of the case hardening of gears made of Pyrowear^®^ 53 steel. The described case can be dangerous, especially for gears utilized in aircraft gearboxes, which must meet stringent reliability demands that translate directly into flight safety.

The gears in aircraft gearboxes usually transfer high power, and the teeth are cyclically exposed to variable loads, which contribute to variable states of stress on the tooth flanks and in the root areas. The variance of the properties of the surface layer, consisting of reduced carbon concentrations in certain areas of the carburized case on the tooth surface, for example, due to the presence of a barrier formed by chips bonded to the same surfaces during carburizing, and which may affect more than just the hardness. It can lead to much more dangerous effects related to extreme pressure, resulting in strain or surface wearing, and hence to failure of the gear or the whole gearbox. The research described further in this paper focuses on presenting the effect of chips which, in the case of a poorly performed tooth forming manufacturing process upstream of the thermochemical processing or limited understanding of the issue, may cause localized reduction in hardness in the areas of interest on the tooth surface, following the processes intended to improve the mechanical performance of a surface layer exposed to high loads.

## 2. Test Material and Methodology

The material chosen for testing was a double helical gear based on Pyrowear^®^ 53 alloy steel (Figure 2) with the concentrations of chemical composition as shown in Table 1 and steel properties shown in Table 2.

**Table 1:** Chemical composition of the PYROWEAR^®^ 53 (AMS 6308) steel [26].

Cr	Ni	C	Mo	Cu	V	Mn	Si	Fe
1.0	2.0	0.11	3.25	2.0	0.1	0.4	0.9	the rest

**Figure 5:**
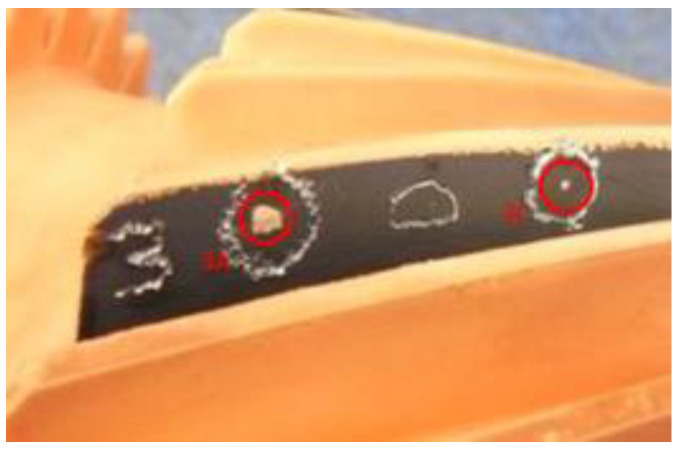
Gear sample with copper “spots” varying in size, representing chips locations close to pitch diameter of the gear teeth.

**Figure 6:**
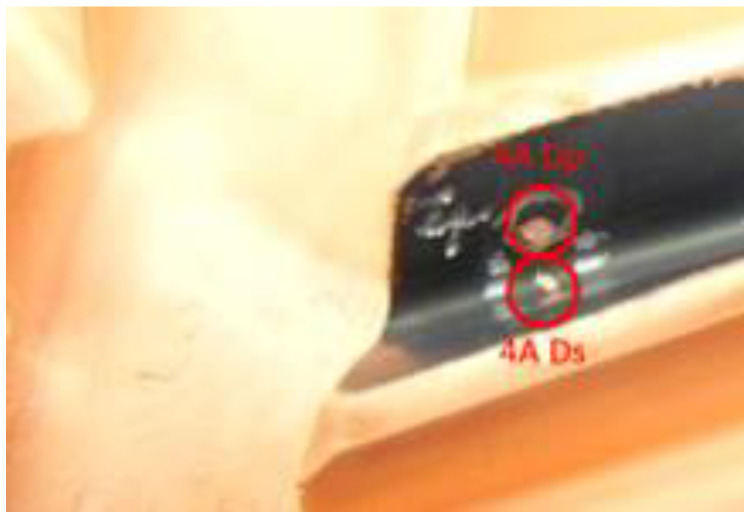
Gear sample with copper “spots” varying in size, representing chips in different locations on the gear teeth.

Prior to tooth cutting by power skiving, the gear was plated with copper, using a galvanic process, to mask the surfaces not to be case-hardened. This operation was intended to accurately represent a typical manufacturing process involving the case-hardening of gears. After power skiving of the gear with a Gleason 600 PS (Gleason-Pfauter Maschinenfabrik GmbH, Ludwigsburg, Germany), it was visually inspected for the presence of chips on the tooth flanks and roots. A ×10 magnifying glass was used as a visual aid. Having confirmed that the gear wheel featured no foreign objects in the form of chips, a number of processes were performed to deposit the copper plating on certain areas to simulate the presence of chips on the tooth surfaces, which was then subject to downstream carburizing and heat treatment. For this purpose, the designated areas of the gear (selected teeth flanks) were protected by paraffin, followed by the selective removal of the paraffin coat to create small holes in specific areas to enable their galvanic copper plating. The described methodology was used to generate copper spots, which simulated the real chips appearing on teeth surfaces. The described technique reliably bound that whole surface of simulated chips to the gear teeth, which would not be possible in the case of using real chips for verification of their influences on the hardened layer. Furthermore, that gave additional assurance about the sizes of the simulated chips. The galvanic copper plating of the test specimen (gear wheel) created selected areas featuring copper “spots” varying in size, representing chips in different locations on the gear teeth (Figure 5 and Figure 6).

The gear wheel was then thermochemically treated based on the parameters specified for Pyrowear^®^ 53 (AMS 6308). The properties of the teeth surface layer were achieved by thermochemical treatment processes, which consisted of the following steps and temperature parameters recommended by the producer: Gas carburizing (870–930 °C), oil quenching/hardening (905–920 °C), subzero treatment (below −75 °C), and tempering (150–290 °C) [2]. The preparation of the test specimens then began. For this purpose, samples were cut from the gear and metallographic specimens were prepared for selected sections, for which the cutting plane was perpendicular to the surface of the areas reflecting the simulated presence of chips. The specimens were etched in Nital 5%, a specialist product for microsections.

**Figure 7:**
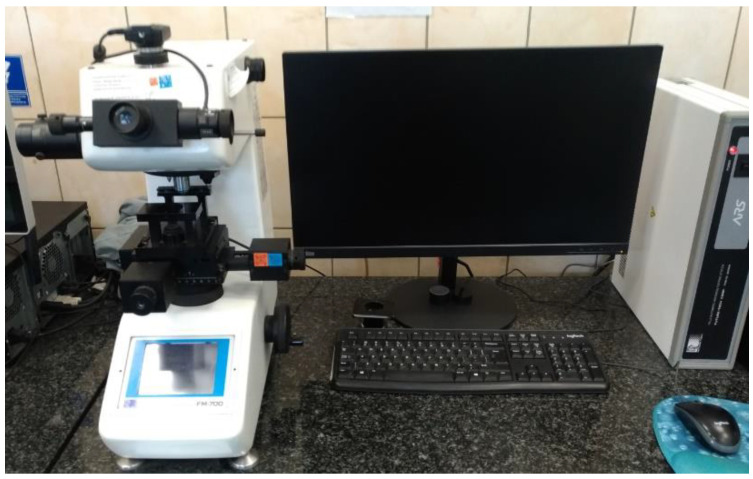
Hardness research station equipped with microhardness tester, Future-Tech Corp., Type: FM-700.

Hardness testing was done with a FM-700 micro-hardness testing machine (Future-Tech Corp., Kanagawa, Japan (Figure 7). The hardness test method was HV_0.5_, ref. ASTM E384-17. The uncertainty for the range 240–600 HV_0.5_ was ±9 HV_0.5_, and for the range >600 HV_0.5_ was ±17 HV_0.5_.

## 3. Test Results and Analysis

The tests provided data on the case hardness values, which enabled an analysis of the effect of chips on the parameter. The tests also allowed visualization of the changes in case hardening depth under large chips over the diffusion case hardened layer. The tests were performed on two characteristic areas of the gear, the pitch diameter and the tooth root. The locations where the chips were simulated by copper ‘spots’ were selected due to the stresses present during typical engagement with the tooth on the mating gear wheel. The simulation was repeated for the cases of a large chip near the pitch circle, as represented by the analytical results for zones 3A and 4A Dp and for the cases of a small chip within the pitch circle (zone 3C) and the tooth flank (4A Ds). The assignment of the chip size to the areas of interest was undertaken with reference to the presence of chips observed during power skiving tests and optimization of power skiving.

**Figure 8:**
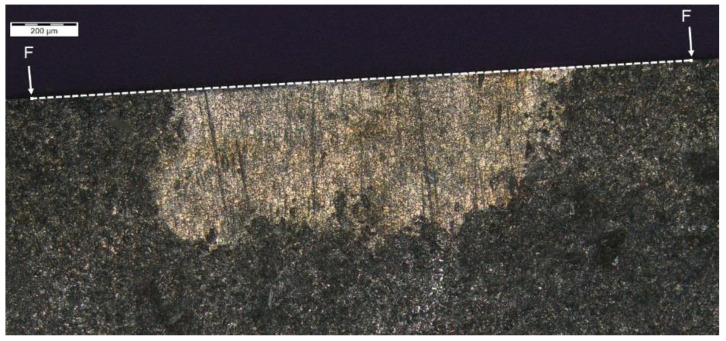
Localization of cross-section F-F, zone: 3A.

**Figure 9:**
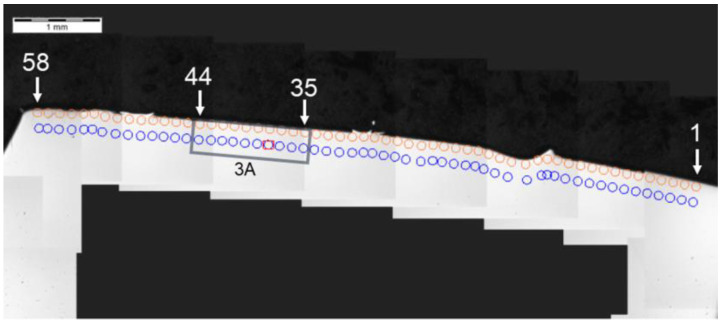
Pattern of hardness verification. Paths depth (distance from tooth surface): 0.1 mm and 0.3 mm. Cross-section F-F, zone: 3A.

**Figure 10:**
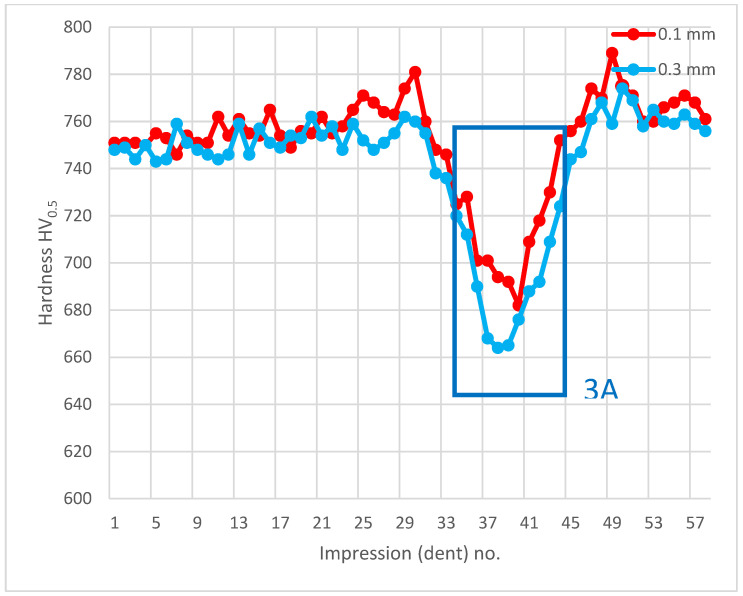
Microhardness measurement results. Paths depth (distance from tooth surface): 0.1 mm and 0.3 mm. Cross-section F-F, zone: 3A.

**Figure 11:**
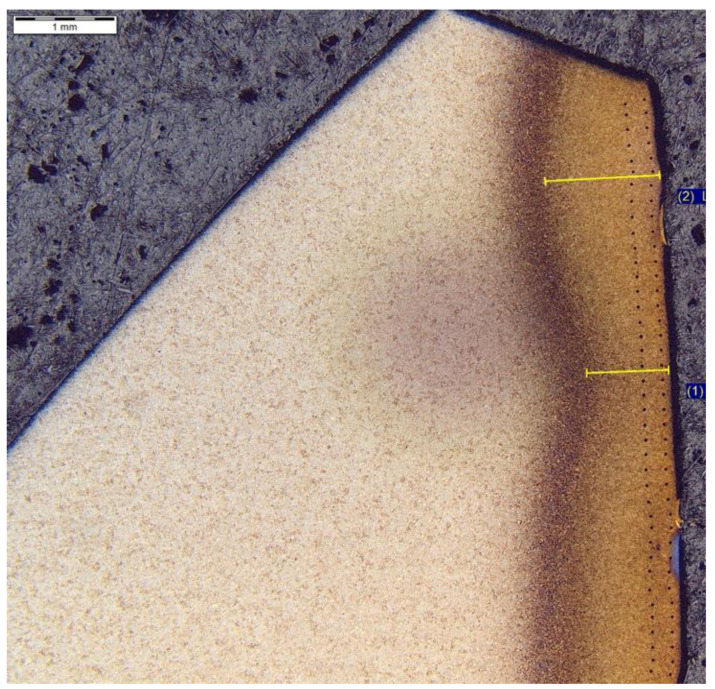
Waviness of carburized case depth. Magnification: 12.5×. Cross-section F-F, zone: 3A. Etched sample—Nital 5%.

The analysis of hardness in chip zone 3A (Figure 5) in cross-section F-F (Figure 8) demonstrated a significant hardness reduction in 3A (Figure 9) in comparison to the adjacent areas. The localized increase in hardness on both sides (Figure 10) were a result of the hardness tests in the zone identification location. Having observed this hardness reduction, tests were performed to image the carburized case depth within 3A. The test showed reduction of carburized case depth in the area of the simulated chip appearing (Figure 11). 

**Figure 12:**
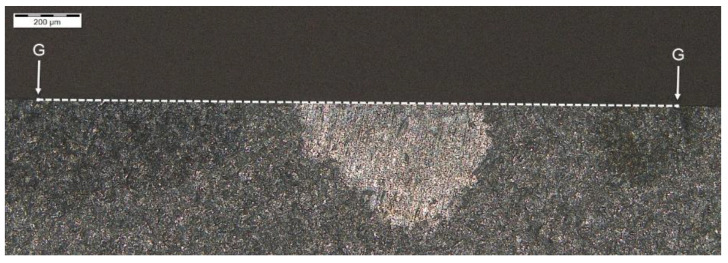
Localization of cross-section G-G, zone: 3C.

**Figure 13:**
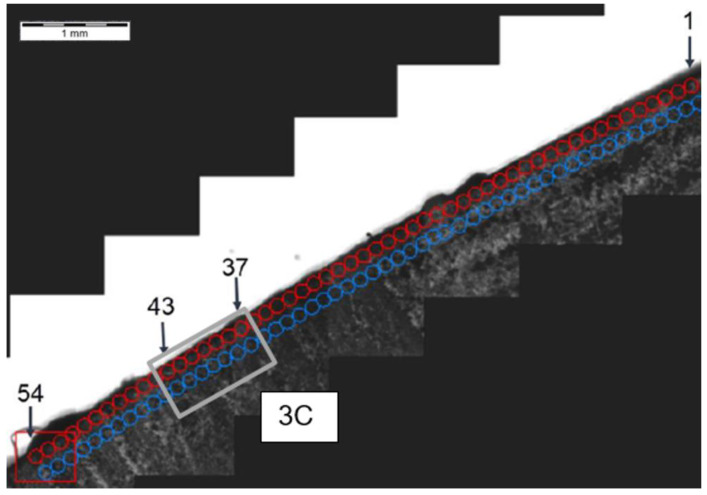
Pattern of hardness verification. Paths depth (distance from tooth surface): 0.1 mm and 0.3 mm. Cross-section G-G, zone: 3C.

**Figure 14:**
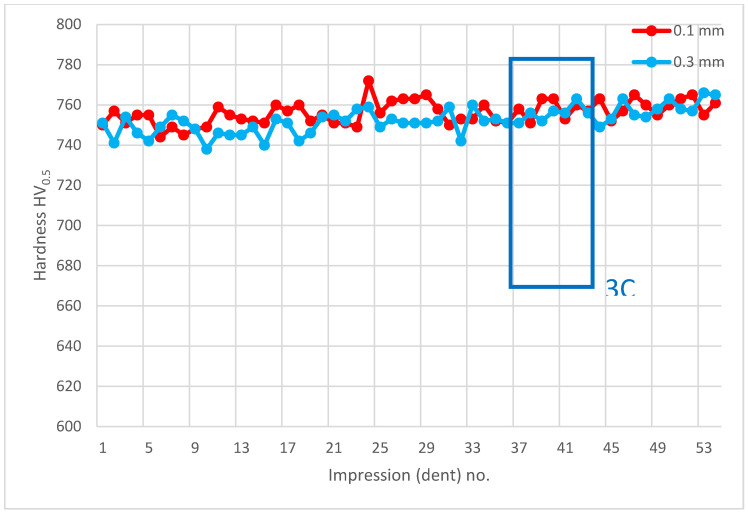
Microhardness measurement results. Paths depth (distance from tooth surface): 0.1 mm and 0.3 mm. Cross-section G-G, zone: 3C.

Another analysis of hardness in chip zone 3C (Figure 5) in cross-section G-G (Figure 12) did not reveal a significant hardness reduction in 3C (Figure 13) in comparison to the adjacent areas (Figure 14). Given this, no further tests were done on the specimen to image the carburized case depth in zone 3C.

**Figure 15:**
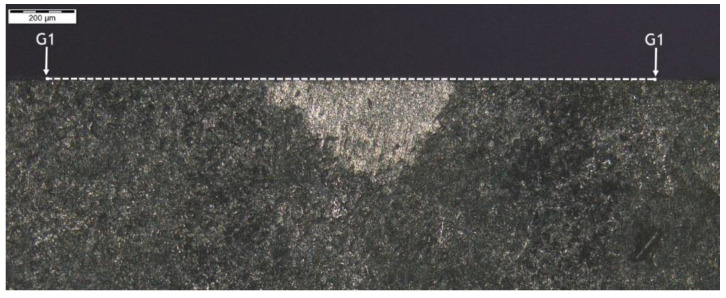
Localization of cross-section G1-G1, zone: 3C.

**Figure 16:**
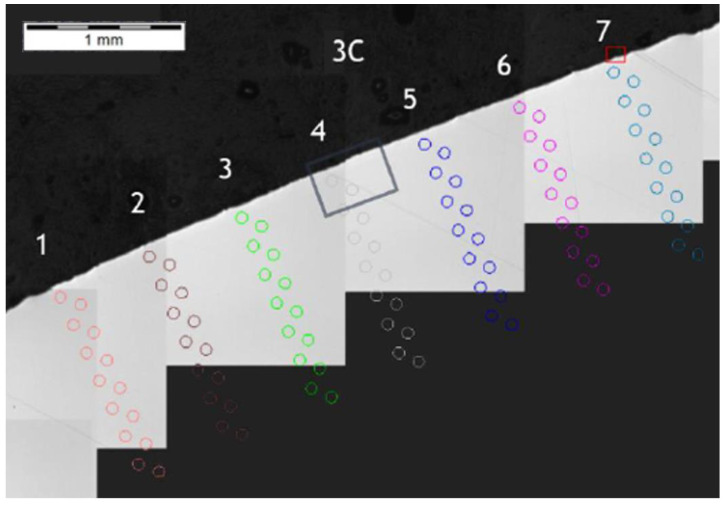
Pattern of hardness verification. Cross-section G1-G1, zone: 3C.

**Figure 17:**
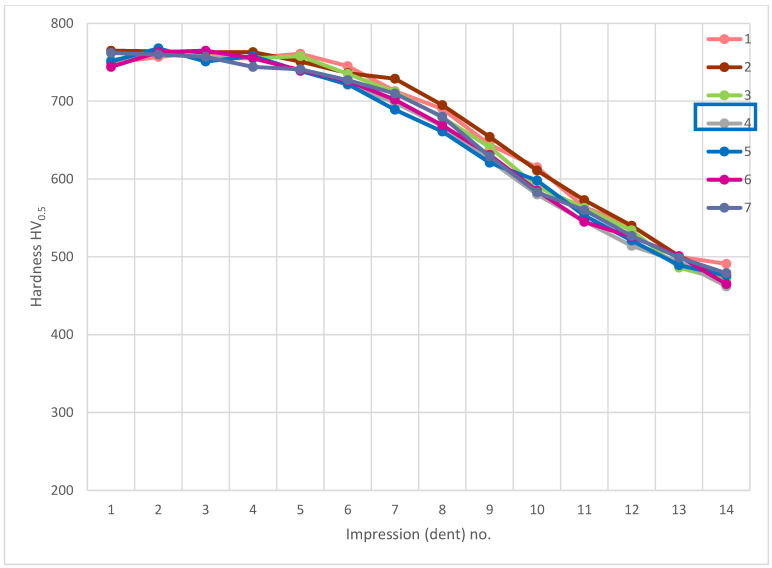
Microhardness measurement results. Cross-section G1-G1, zone: 3C.

Another microsection of zone 3C was made as cross-section G1-G1 (Figure 15) and had a hardness distribution analysis was performed perpendicular to the outer surface of the surface layers, as shown by the measurement results in Figure 16. The hardness distribution shown for pattern (path) 4 (Figure 17) did not deviate from other hardness distribution patterns in the area of interest and which indicates a negligible or lack of influence of small chips on the effect of carbon diffusion or hardness after thermochemical treatment adjacent to the pitch circle.

**Figure 18:**
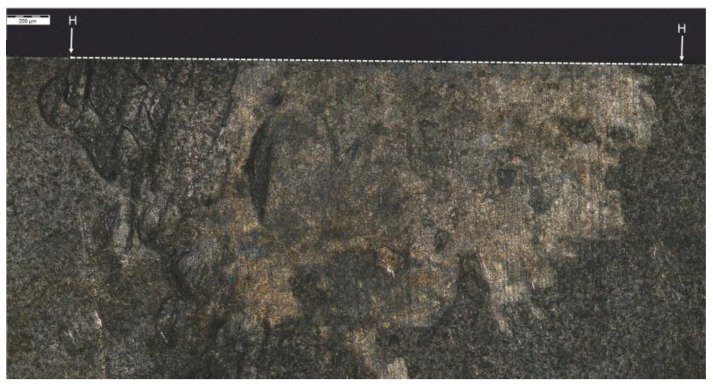
Localization of cross-section H-H, zone: 4A Dp.

**Figure 19:**
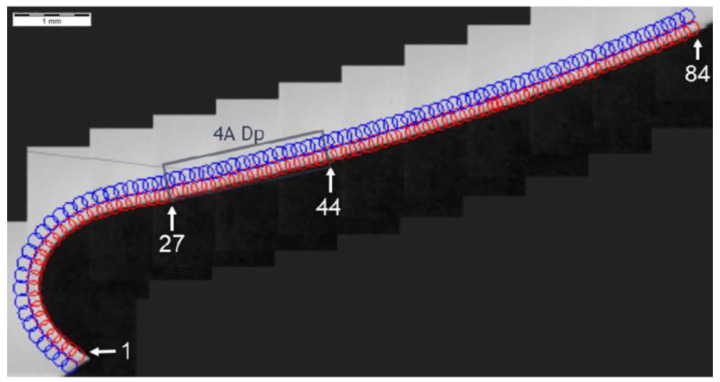
Pattern of hardness verification. Paths depth (distance from tooth surface): 0.1 mm and 0.3 mm. Cross-section H-H, zone: 4A Dp.

**Figure 20:**
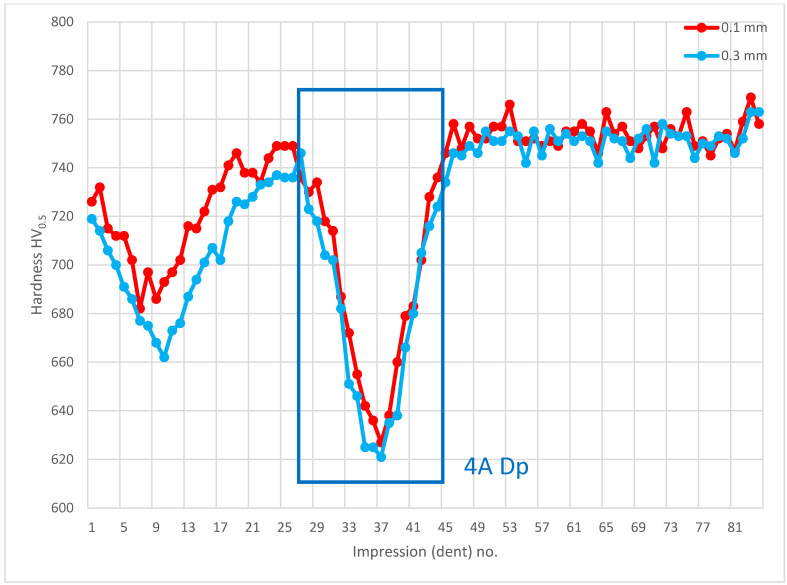
Microhardness measurement results. Paths depth (distance from tooth surface): 0.1 mm and 0.3 mm. Cross-section H-H, zone: 4A Dp.

**Figure 21:**
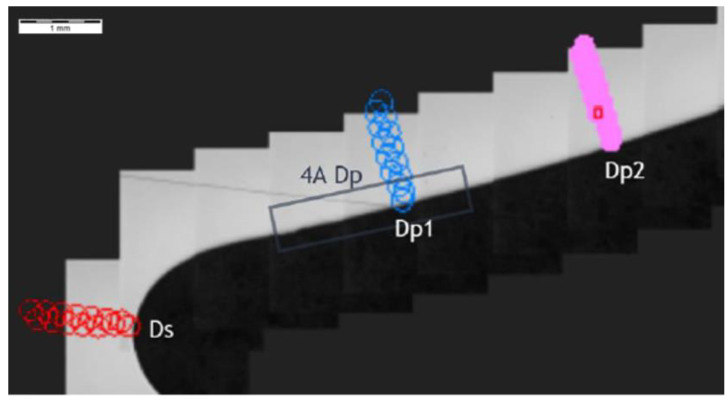
Pattern of hardness verification. Cross-section H-H.

**Figure 22:**
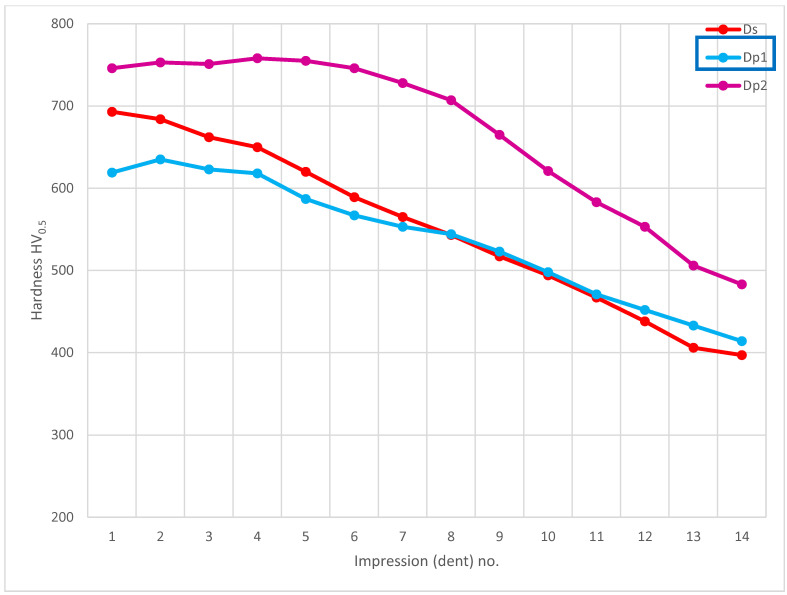
Microhardness measurement results. Cross-section H-H.

**Figure 23:**
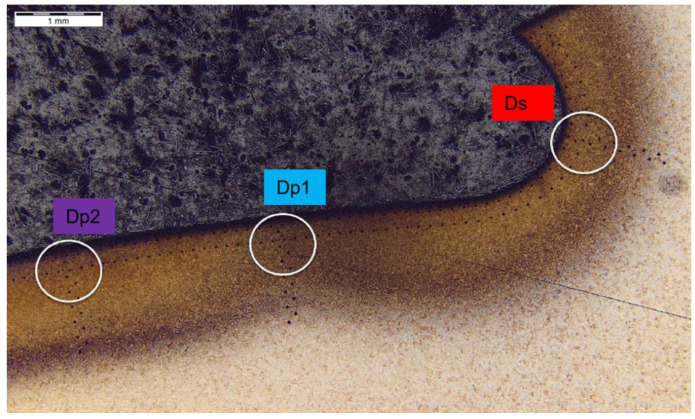
Waviness of carburized case depth. Reduction in carburized case depth within the area Dp1. Magnification: 12.5×. Cross-section H-H. Etched sample—Nital 5%.

Further, the data for the chip presence in zone 4A Dp (Figure 6) for cross-section H - H (Figure 18) was analyzed and demonstrated a significant hardness reduction in 4A Dp (Figure 19) in comparison to the adjacent areas, as illustrated by the hardness distribution pattern (Figure 20). For 4A Dp, a hardness distribution analysis was performed perpendicular to the outer surface of the carburizing layer, as shown by the measurement pattern (path) in Figure 21. The hardness distribution pattern for the Dp1 set (Figure 22) markedly deviated from the hardness distribution pattern of the Dp2 pattern (path), the spread of which was typical for a normal distribution hardness, and formed a control (crosscheck test) for this test. The analysis of the pattern (path) revealed the significant effect of chips on carbon diffusion and ultimately the case hardness after thermochemical treatment. To better illustrate the effect of the chip on the carburized case, additional tests were performed to image the carburized case depth in zone 4A Dp, the results of which are shown in Figure 23 as a distinct reduction in carburized case depth within the area of the chip, as shown by the Dp1 pattern (path). The hardness distribution chart (Figure 20) shows another sector with an observable case hardness reduction, including two measurement points within the tooth root area. Its interpretation is shown further later.

**Figure 24:**
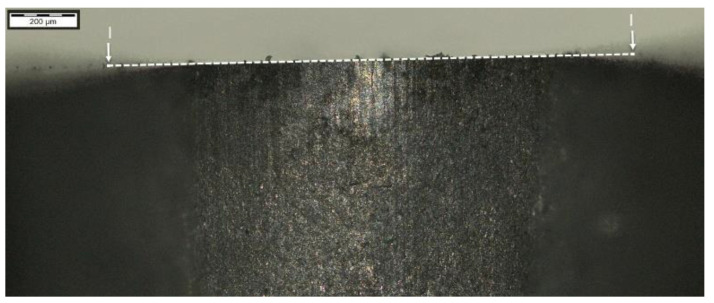
Localization of cross-section I-I, zone: 4A Ds.

**Figure 25:**
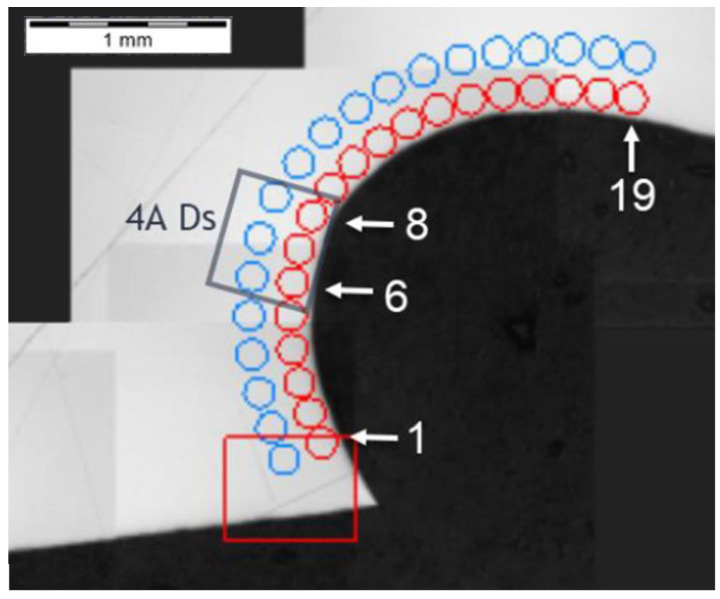
Pattern of hardness verification. Paths depth (distance from tooth surface): 0.1 mm and 0.3 mm. Cross-section I-I, zone: 4A Ds.

**Figure 26:**
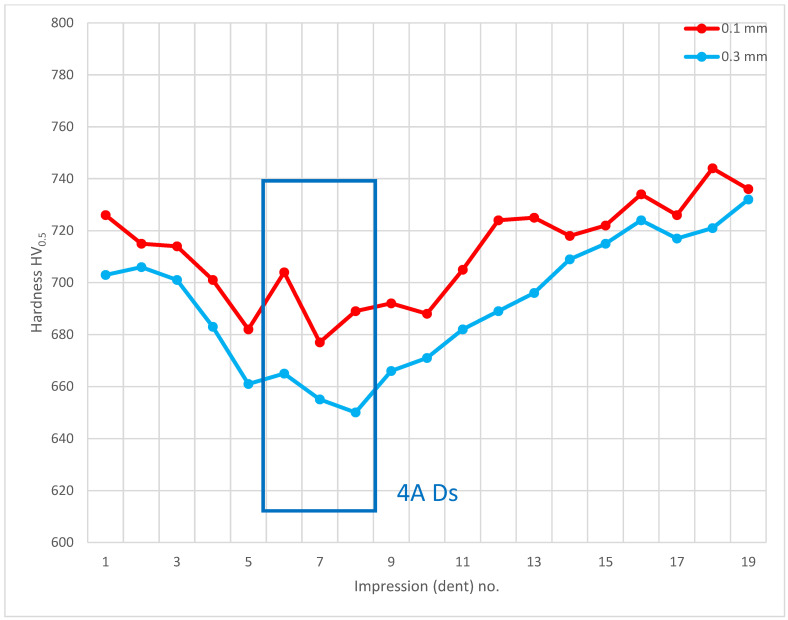
Microhardness measurement results. Paths depth (distance from tooth surface): 0.1 mm and 0.3 mm. Cross-section I-I, zone: 4A Ds.

The last of the processed areas of interest was 4A Ds (Figure 6), representing the tooth root area. The tests were performed on cross-section I-I (Figure 24). The testing revealed a hardness reduction in 4A Ds (Figure 25), as demonstrated by the hardness distribution pattern (Figure 26). For zone 4A Ds, the hardness distribution pattern showed a reduction in hardness, but it must be noted that here the tooth root area is a region where, due to the geometry of the gear and its adjacent teeth, the kinetics of the carbon diffusion into the surface layer is not as efficient as in the areas near the pitch circle, for example. The effect can be observed in Figure 20, at those points located next to zone Ds, where subsequent measurements proved that the hardness grew with the distance from the tooth root. This effect was also observed in the detailed analysis shown in Figure 23, where the region near zone Ds featured an observable change of the carburized case depth in comparison to zone Dp2, which was a control for all tests on the specimen. Note, however, that with a small chip no distinct effect on the case hardening depth was observed, while the small, localized reduction in hardness might not only be an effect of the chip, but also a lower concentration of carbon from inhibited diffusion during carburizing, or possibly a slight contribution from an error in the measurement method.

## 4. Conclusions

Tests demonstrated that chips pressure welded or bonded to the surfaces of the gear teeth made of Pyrowear^®^ 53 subject to downstream thermochemical treatment can negatively affect the process of carburizing. Under certain circumstances, the outcome of this effect may cause a localized reduction in hardness of the surface (carburized case) layer. Consequences of the unfavorable impact are particularly dangerous when the copper from the coating described in the test methodology is attached to the tooth surface along with the steel chip, which forms a barrier to the carbon diffusion process and is cut together with the chip during its formation. This phenomenon is extremely hazardous to gears working under high loads, where stress concentrations can lead to the failure of the gear or the entire gearbox.

Tests revealed that small chips (size under 1.5 mm) do not have a negative effect on the case hardness in the area of the pitch circle but, given the geometry of the teeth and the decelerated kinetics of carbon diffusion within the tooth root, the chips present in this part of the gear could result in small, localized reductions in hardness. The larger the size of chip (size over 1.5 mm) present on a tooth surface during the carburizing process, the more it can intensify the unfavorable localized reduction in carburized case depth, reduction in carbon concentration in the surface layer obscured by the chips, and localized reduction in hardness for those areas obscured by the chips. Given this, it is crucial to properly implement and optimize power skiving upstream of any thermochemical processing, as the impact of chips on tooth surfaces or other areas processed by thermochemical treatment may negatively affect the performance of the components. It is prudent to improve the machining processes to a point where chip fusion or bonding does not occur. The phenomenon discussed in this work is not only related to power skiving of gear teeth. It can also be present in Fellows slotting and other machine cutting processes. It is important that effects of similar nature do not occur or are eliminated at the machining stages, especially for components which feature areas processed by carburizing. This will help to avoid problems related to localized case hardness reduction in the finished products, and operating issues with these products.

## Figures and Tables

**Table 2:** PYROWEAR^®^ 53 (AMS 6308) steel properties (typical) [27].

Alloy	Yield Strength	Ultimate Tensile Strength	Core Hardness	%EI	%RA	K_IC_ Toughness	Achievable Surface Hardness	Tempering Temperature
Pyrowear® Alloy 53	965 MPa (140 ksi)	1172 MPa (170 ksi)	354–434 HV (36–44 HRC)	16	67	126 MPa × √m (115 ksi × √in)	674-722 HV (59–63 HRC)	204 °C (400 °F)
Hardness conversations from HRC to HV per ASTM E140								

## Data Availability

Data is contained within the article.
